# Daniel Aberdam, the legacy of a mentor

**DOI:** 10.1038/s41418-025-01520-9

**Published:** 2025-06-03

**Authors:** Huiqing Zhou, Bernard Attali, Neil Lagali, Ruby Shalom-Feuerstein

**Affiliations:** 1 Department of Molecular Developmental Biology, Faculty of Science, Radboud Institute for Molecular Life Sciences, Radboud University, Nijmegen, the Netherlands; Department of Human Genetics, Radboud University Medical Center, Nijmegen, the Netherlands; 2 Department of Electrophysiology, School of Medicine, Tel Aviv University, Tel Aviv Israel; 3 Division of Ophthalmology, Department of Biomedical and Clinical Sciences, Linköping University, Linköping, Sweden; 4 Department of Genetics & Developmental Biology, The Rappaport Faculty of Medicine & Research Institute, Technion Integrated Cancer Center, Technion - Israel Institute of Technology, Haifa, Israel

**Keywords:** Gene regulation, Desmosomes

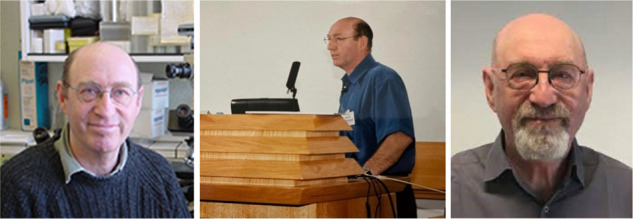



Daniel Aberdam (1954–2025)

*If I should go tomorrow It would never be goodbye, For I have left my heart with you (Anon)*

It is with profound sadness that we announce the passing of Daniel Aberdam, a distinguished French and Israeli scientist, on April 7, 2025. Daniel was a brilliant researcher whose contributions to the fields of skin and corneal research have left a substantial mark on the scientific community. Among his many achievements, Daniel’s research on the role of Laminin 5 and integrin β4 mutations in junctional epidermolysis bullosa, and his pioneering studies on modeling skin and corneal lineage commitment and pathophysiology using pluripotent stem cells stand out. In the years leading up to his passing, Daniel established and promoted international collaboration focused on developing and testing novel therapies for skin and corneal pathologies using small molecular weight compounds. His collaborative spirit ensures that his research will continue, as his friends and colleagues are committed to advancing his innovative ideas. Daniel’s legacy will live on through the ongoing work of those he inspired and mentored. These collaborations are in progress, ensuring that his innovative research continues to evolve and make an impact.

Daniel received his PhD from the Weizmann Institute of Sciences (Rehovot), where he studied the oncogenic potential of homeobox genes in leukemia under the mentorship of Prof. Leo Sachs. As a postdoc under the supervision of Prof. Jean-Paul Ortonne, Daniel uncovered the importance of Laminin-Integrin interactions to skin biology and discovered that point mutations in either of these interacting proteins result in epidermolysis bullosa syndrome, a congenital skin blistering disease. His work helped establish the relationship between specific genetic mutations and clinical manifestations of the pathology that mutations in these proteins disrupt the connection between the epidermis and dermis, delineating the various types of blistering diseases. Moreover, it advanced the accurate diagnosis, and allowed more suitable treatment for patients.

In 1995, Daniel was recruited by INSERM as Director of Research, where he led a research unit at the University of Nice Sophia Antipolis (Nice). A couple of years later, Daniel established the unique French-Israeli binational team at the Technion (Haifa). During those years, his work pioneered developing new protocols for generating skin and corneal cells from embryonic stem cells and later induced pluripotent stem cells (iPSC). His teams developed pluripotent stem cell-based cellular models for studying the genetic pathways involved in embryonic cell fate decisions and developing patient-specific therapies. These studies encompassed various aspects, from early embryogenesis and exit from pluripotency, to ectodermal commitment into neural, epidermal, and corneal lineages. They illuminated the roles of morphogens, transcription factors, and microRNA genes essential for skin and corneal development, as well as stem cell self-renewal and differentiation. In the last decade, Daniel returned to his beloved city, Paris, where he continued his research, focusing on developing novel therapeutic approaches for skin and corneal diseases. He was particularly excited about the promising effects of the small molecular weight compound PRIMA-1^MET^ in clinical trials for ectodermal dysplasia patients with mutations in the key epithelial stem cell transcription factor, p63. Encouraged by these outcomes, he further explored new therapies for corneal stem cell deficiency diseases, including aniridia. In recent years, Daniel’s research made an indelible mark on the aniridia research community, where he pioneered drug library screening to discover therapeutic compounds for disorders related to the transcription factor PAX6. His team identified approved compounds for drug repurposing, including duloxetine/Cymbalta, demonstrating its potential to rescue PAX6 haploinsufficiency in vitro, which has since been confirmed by others in vivo. He was passionate about the work with PAX6, with his team developing a CRISPR-Cas9 gene-edited corneal stem cell line with PAX6 mutation as a research tool, that he generously shared with researchers across Europe and is now actively used in many laboratories. His leadership within European projects and networks on aniridia led to collaborations and scientific exchanges training a new generation of scientists. His work in this field is actively being pursued by multiple research teams and will undoubtedly lead to further advances in the near future.

Daniel was a brilliant scientist, a close and beloved friend, and an inspiring mentor for many scientists. His vibrant personality, passion for research, and love for people made him a unique, warm, and cherished individual. His infectious enthusiasm connected and inspired everyone around him. He was kind-hearted, generous with praise and encouragement, and deeply loved by nearly all who knew him. Daniel was a man of many ideas, passionate for the quest of truth, justice, and ethics. He was also full of contradictions—optimistic yet sometimes pessimistic, patient yet often impatient, but always with a charming grace, sense of humor and extremely generous personality.

After being diagnosed with pancreatic cancer in February 2024, Daniel spoke openly about death while bravely seeking cure, with faith in science, fully aware of its limitations. After his diagnosis and during treatment, Daniel continued to collaborate, exchange ideas and mentor others by teleconference, above all continuing to spread his enthusiasm and love of science. He succumbed to the illness on April 6, 2025. Daniel’s work has been widely published in prestigious journals, and his legacy will continue to inspire future generations of scientists. He also had a relevant role in scientific publishing, both for *Cell Death Differentiation* and for *Stem Cells*, continuously inspiring and supporting all scientists.

Daniel will remain in our hearts as an extraordinary model of humanity and dedication. We will greatly miss his curiosity and intelligence, his passion for research, his availability and kindness with family, friends, collaborators as well as with competitors. We miss him dearly and will always remember him as a colorful, passionate, and loving person whose legacy will live on in the hearts of those he touched and in the scientific community he so fervently believed in. Our hearts remain close to Edith, his children and grandchildren.

